# *“I haven’t had that information, even though I think I’m really well-informed about most things”*: a qualitative focus group study on Australian women’s understanding and views of potentially modifiable risk factors for breast cancer

**DOI:** 10.1186/s12905-023-02363-7

**Published:** 2023-04-28

**Authors:** Brooke Nickel, Josephine Armiger, Christobel Saunders, Wendy Vincent, Rachael H Dodd, Anthea Temple, Nalini Bhola, Angela Verde, Nehmat Houssami

**Affiliations:** 1 Sydney Health Literacy Lab, School of Public Health, The University of Sydney, Camperdown, NSW Australia; 2 Wiser Healthcare, School of Public Health, The University of Sydney, Camperdown, NSW Australia; 3 Department of Surgery, University of Melbourne, Victoria, Australia; 4 BreastScreen NSW Sydney Local Health District, Royal Prince Alfred Hospital, Camperdown, NSW Australia; 5 The Daffodil Centre, A joint venture between Cancer Council NSW, Faculty of Medicine and Health, The University of Sydney, The University of Sydney, Camperdown, NSW Australia; 6 BreastScreen NSW, Cancer Institute NSW, Camperdown, NSW Australia; 7 Breast Cancer Network Australia, Victoria, Australia

**Keywords:** Breast cancer, Risk factors, Communication, Lifestyle, Qualitative

## Abstract

**Background:**

Building health literacy about potentially modifiable risk factors for breast cancer may help to empower women to make more informed decisions about their breast health; however there has been limited qualitative research on this topic. This study aimed to explore current knowledge, understanding and experience of potentially modifiable risk factors for breast cancer, and views on current and future communication strategies for this information and related interventions.

**Methods:**

Qualitative study using online focus groups via Zoom in October-November 2022. A diverse sample of women from the Australian community aged 40–74 years were recruited.

**Results:**

Fifty-one women from a range of socioeconomic backgrounds took part in nine focus groups. General knowledge of risk factors for breast cancer in the community is limited, particularly in relation to modifiable factors such as alcohol consumption and postmenopausal obesity, with many women describing feelings of ‘shock’ following this information. Women overwhelming believed that information on modifiable risk factors for breast cancer should be communicated more widely, however communication preferences for receiving this information varied. There was a strong preference amongst the women for a cascade of information which they believed may then help target greater number of women of all ages and backgrounds. Despite worry about long-term compliance, women also supported various lifestyle interventions which may help them and other women to reduce their overall risk.

**Conclusions:**

Findings from this study highlight the need for more widespread community communication and education about risk factors for breast, in particular potentially modifiable risk factors such as alcohol consumption and postmenopausal obesity. As breast screening programs in Australia and globally begin to evaluate the potential for risk-related screening this will provide an additional context for primary prevention, hence planning of messaging and piloting of lifestyle-related prevention strategies in breast cancer is needed now. Gaining an understanding of women’s preferences for communication and forms of interventions is vital to ensure their engagement.

## Background

Breast cancer is the most commonly diagnosed cancer among women in Australia, and the second most commonly diagnosed cancer in Australia [1]. Although breast cancer mortality rates have decreased in recent decades, breast cancer remains one of the leading causes of cancer-related disease burden among women [2]. Thus, breast cancer prevention is an important strategy to reduce the risk and potential burden of breast cancer among women [3].

Breast cancer risk factors vary over a woman’s lifespan. In women around the age of menopause and older, relatively modifiable risk factors for breast cancer (e.g. postmenopausal obesity, high alcohol consumption) carry similar risks to non-modifiable more well-known risk factors such as family history [4]. Epidemiological studies have estimated that 30–50% of postmenopausal breast cancer cases can be prevented through modifying lifestyle behaviours [4, 5]. Significant breast cancer risk reductions are achievable through starting lifestyle change around and after menopause, additional to the often-emphasised life course approach to breast cancer prevention [4]. It has been reported that women lack awareness of lifestyle change as a form of breast cancer prevention.[6] Greater public awareness is evident for risk management strategies such as screening, self-examination, and surgical strategies such as prophylactic mastectomy [6]. When asked about lifestyle change as a form of breast cancer risk management, women have generally been open to receiving advice [7, 8].

While knowledge of risk factors does not guarantee actual lifestyle change – as demonstrated by evidence in the field of chronic disease research [9] – awareness and understanding of the link between risk factors and breast cancer risk has been identified as a key factor in the uptake of lifestyle change for breast cancer risk management [7]. Women are interested in receiving information about their risk of breast cancer [6], however the views of Australian women on the types of communication and delivery of lifestyle interventions are largely unknown.

Building health literacy about risk factors for breast cancer aligns with the proposed Cancer Plan for Australia [10]. It may help to empower women to make more informed decisions about their own breast health. Furthermore, it may potentially change behaviour around modifiable risk factors; inform the development of population prevention strategies [11]; and help improve cancer outcomes, and overall health, given commonality of risk factors across preventable chronic disease.

This study aimed to explore current knowledge, understanding and experience of potentially modifiable risk factors for breast cancer, and views on current and future communication strategies for this information and interventions among women living in the Australian community.

## Methods

### Study design

A qualitative study using online focus groups was conducted which provided structured information about breast cancer risk factors via a presentation, interspersed with group discussions and opportunities for questions.

The study was approved by the University of Sydney Human Research Ethics Committee (2022/282). Participants were sent an email containing the participant information sheet and the consent form, which was digitally signed prior to participating in the focus group.

### Participant recruitment

A community sample of women across Australia aged 40–74, who are approaching menopause or are post-menopausal were recruited. Women in this age group are either invited for biennial mammograms as part of the publicly funded screening program BreastScreen (aged 50–74), or are eligible to attend BreastScreen for a mammogram (aged 40–49). While it is recognised that there is no consensus on the balance of benefits and harms of screening women aged 40–49 and hence no recommendations in this age-range differs across countries, for the purpose of this study all women who are potentially screened through the BreastScreen Australia program were included. Recruitment was carried out through an independent research recruitment organisation (Taverner Research), who used random digit dialling and social media advertising to approach potential participants. Trained interviewers telephoned potential participants with a pre-recruitment eligibility check to ensure the participant fulfilled the recruitment criteria and that they had adequate equipment (laptop or desktop computer, and access to Zoom) to participate in the focus groups. Women were excluded if they had had a previous diagnosis of ductal carcinoma in-situ or breast cancer, did not speak adequate English, or did not have the capacity to consent.

To gain a diversity in perspectives in the focus groups, quota sampling was used to ensure inclusion of participants with varying levels of education and health literacy backgrounds. The study aimed to recruit 25% of participants who came from culturally and linguistically diverse backgrounds (CALD) (e.g., who spoke a language other than English at home but spoke English well enough to participate in the focus group), which reflects the 22.8% of Australians who speak a language other than English at home [12].

Focus groups were stratified across three age categories: 40–49; 50–59; 60–74. Three focus groups were run for each age group, totalling nine focus groups altogether, with the aim of recruiting six women for each focus group in order to optimise group dynamics – ensuring everyone was able to participate while having enough people to keep the conversation flowing. This number of participants also facilitated thematic saturation, as indicated by data redundancy (e.g., when participants no longer raised original themes). An additional pilot focus group was run with five women aged 40–74.

Each participant was given a $100 gift voucher at the conclusion of the focus group as compensation for their time and any costs associated with attendance.

### Focus group presentation and discussion

Online focus groups were conducted via Zoom across October-November 2022. An additional pilot focus group was conducted in October 2022 to trial the presentation material with the study population. Minimal changes were made, namely to questions posed to participants to improve the flow of the presentation and focus discussions.

Each focus group lasted approximately one and a half hours, and consisted of an introduction, a demographic questionnaire, a PowerPoint presentation interspersed with discussions and questions, and a final questionnaire regarding knowledge and attitudes towards breast cancer risk factors. Questionnaire links were sent in the Zoom chat during the focus group session, data was collected using Qualtrics.

Each focus group was facilitated by one female moderator (BN) with a PhD in public health, alongside another female moderator (JA) with a Masters of Public Health who addressed participant technical queries and took notes. BN gave an audio-visual presentation at each focus group, which had been developed and reviewed by a multidisciplinary team of public health and medical researchers, breast clinicians, a health psychologist, BreastScreen staff, and a consumer representative. It was developed using high-quality and up-to-date research evidence interpreted for a lay audience [13–18]. The presentation consisted of plain language text, graphs, and images. It presented information about breast cancer screening, risk factors, sources and communication methods of information about breast cancer risk factors, and potential methods of lifestyle interventions for addressing breast cancer risk factors. See Table 1 for the topic guide and key discussion questions (the complete presentation is available from authors upon request).

At the beginning of each focus group, it was made clear that study researchers were not looking for a consensus in the discussions and that it was important to hear everyone’s honest thoughts and opinions. It was stated that disagreements were normal to have but asked that respect be maintained between all members of the group and moderators.

**Table 1:** Focus group presentation topics and key discussion questions

PowerPoint presentation content	Corresponding questions for discussion
Breast cancer overview • What is breast screening • How is breast cancer diagnosed and managed	• Do you understand this information? • What are your previous experiences with breast screening?
Breast cancer risk factors • What is a risk factor • Non-modifiable vs. modifiable risk factors • Pre/post-menopausal risk factors • In-depth discussion of some risk factors (age, family history, breast density, obesity, alcohol)	• What are your thoughts on this information? • Is any of this information new? • How does it make you feel? • Is there anything that worries you about this information? • Have you ever had your personal breast cancer risk factors assessed? Why/why not? • Do you think this information should be communicated to women? Why/why not?
Risk factor information • Breast cancer risk information presentation types (e.g. written, visual, spoken, etc.) • Breast cancer risk information sources (e.g. website, GP, etc.)	• What are your thoughts on this information? • How do you think you would like breast cancer information presented to you? • How do you think you’d like to receive this information (from whom and in what format)?
Lifestyle interventions • Different kinds of lifestyle interventions to reduce risk factors for breast cancer	• Are there any lifestyle interventions to reduce breast cancer risk that you would be interested in getting more personalised support towards if relevant to you? • If so, which ones, and which methods of support do you think you’d prefer? • Who would you prefer to receive support from?
Communication • Potential future communication strategies	• Do you have any further thoughts or suggestions about communication about breast cancer risk factors?

### Data collection and analysis

Focus group discussions were audio recorded via a recording device external to Zoom, transcribed verbatim, and analysed thematically. The analysis initially took an inductive approach to ensure that the findings were grounded in participant responses and the data was analysed at semantic level. Notes were initially made during the focus group by one researcher. Alongside these, two researchers independently read the transcripts and developed an initial list of recurring themes. Using constant comparison, the two researchers looked for similarities and differences in the data and the initial coding framework was developed and discussed. Once the coding framework was finalised, coding was performed in NVivo 11 one researcher and checked by the second, with slight modifications made throughout and discussed. The final coding was then examined to identify overarching themes and concepts.

Questionnaires were administered at the beginning and end of each focus group to capture participant characteristics and provide additional quantitative data to contextualise the qualitative data, using survey questions adapted from previous literature and purpose-designed questions. The initial questionnaire collected sociodemographic measures, along with self-rated health, family history of cancer, screening history, cancer history, breast cancer worry [19], and wellbeing [20]. The final questionnaire captured key aspects of understanding and attitudes towards information covered in the presentation through multiple choice questions and free-text responses. Questionnaires were administered using Qualtrics, and the quantitative results were analysed using SPSS.

## Results

Table 2 outlines the participant characteristics of the 51 women from a range of socioeconomic backgrounds who took part in the nine online focus groups.

**Table 2:** Participant characteristics (n = 51)

Characteristic	No. of participants, n (%)
Age 40–49 50–59 60–74	17 (33.3) 17 (33.3) 17 (33.3)
Marital status Married/living with a partner Divorced/separated Widowed Single	23 (45.1) 15 (29.4) 3 (5.9) 10 (19.6)
Aboriginal or Torres Strait Islander Aboriginal Torres Strait Islander	1 (2.0) 1 (2.0)
State Australia Capital Territory New South Wales Queensland South Australia Tasmania Victoria Western Australia	6 (11.8) 17 (33.3) 9 (17.6) 7 (13.7) 2 (3.9) 9 (17.6) 1 (2.0)
Birthplace Australia Europe (Italy, Germany, UK) Asia (India, Indonesia, Hong Kong) Other (Egypt, Monaco, New Zealand, Papua New Guinea, USA)	35 (68.6) 6 (11.8) 5 (9.8) 5 (9.8)
Years since moving to Australia (if born overseas) 10–20 20–40 > 40	4 (7.8) 4 (7.8) 7 (13.7)
Language spoken at home English only English and another language at home	38 (74.5) 13 (25.5)
Highest educational qualification University degree Diploma or certificate HSC or leaving certificate (or equivalent) School certificate or intermediate certificate (or equivalent) No school or other qualifications	27 (52.9) 14 (27.5) 6 (11.8) 3 (5.9) 1 (2.0)
Employment status Full time Part Time Retired Studying or other	16 (31.4) 19 (37.3) 9 (17.7) 7 (13.7)
Rurality Major city Inner regional Outer regional	36 (72) 10 (20) 4 (8)
General self-rated health Excellent, very good, good Fair, poor	38 (74.5) 13 (25.5)
Family history of breast cancer Yes No Unsure	17 (33.3) 33 (64.7) 1 (2.0)
Known personal BRCA-1 or BRCA-2 mutation (if family history of breast cancer) Yes No Unsure	0 (0) 4 (7.8) 13 (25.5)
Breast screening history Never Once Twice Three times Four or more times	11 (21.6) 11 (21.6) 6 (11.8) 3 (5.9) 19 (37.3)
Time since last screen (if screened) Within the last 2–3 years 3 years ago or more	32 (62.8) 8 (15.7)
Breast cancer worry Not worried at all A bit worried Quite worried Very worried	12 (23.5) 33 (64.7) 4 (7.8) 2 (3.9)
WHO-5 Wellbeing Index Score^b^ (Mean (SD))	56.4 (22)

^a^Calculated using the ARIA lookup tool [21]

^b^A score of 0 represents the worst possible wellbeing and 100 represents the best possible wellbeing [20]

The qualitative analysis identified four main themes. Participant quotes are presented throughout the results to illustrate common and diverse responses. There were no distinct differences observed between age groups, however outlined are a few points where age considerations were relevant.

### Limited general knowledge about risk factors for breast cancer

In general, there was poor knowledge about both non-modifiable and modifiable risk factors for breast cancer among women in the focus groups. Women discussed their knowledge about the importance of screening in relation to preventing breast cancer and that this was predominately the information they had seen or heard about prevention, but admitted they knew very little about risk factors in relation to primary prevention.

Some women had heard of the link between family history and breast cancer, however women were unaware of specific information relating to this risk. This was also true for women who stated they had a known family history of breast cancer.

Other non-modifiable risk factors including age and breast density were not well-known with only a few women having previous knowledge on these risk factors. There was an overall knowledge amongst the women that some risk factors tend to increase with age, yet women did not realise that there was a marked increase in risk of breast cancer related to increasing age. Breast density had only been heard about by a few women across the focus groups.

*“One thing that was new to me was the sharp incline in likelihood [of breast cancer] as women age. I didn’t realise that. And understanding that there’s a lot of the risks that you can’t control. You can’t control your age, you can’t control the breast density. Actually, I don’t think I knew about breast density.”* (FG3, 40–49 yrs).

The majority of women in the focus groups also did not know that lifestyle (or modifiable) factors could increase one’s risk of breast cancer. While some women knew about certain risk factors such as smoking for lung cancer or sun exposure for melanoma, they were unaware about specific modifiable risk factors for breast cancer.

*“Some of the lifestyle factors in a way surprised me … of course we all think of smoking/lung cancer, things like that, but sometimes I don’t necessarily associate those lifestyle things with breast cancer or possible other cancers as well.”* (FG2, 40–49 yrs).

### Shocked about information on modifiable risk factors

The information presented to women on modifiable risk factors, specifically alcohol consumption and postmenopausal obesity, generated a lot of conversation amongst the women. This was mainly around the novelty of the information with most women never having heard that these factors were linked to risk of breast cancer.

*“I didn’t actually realise about the alcohol. I don’t think I’ve ever seen anything about risk factors and alcohol”* (FG7, 60–74 yrs).

*“I am quite shocked about the weight thing, and that I haven’t had that information, even though I think I’m really well-informed about most things.”* (FG8, 60–74 yrs).

Women discussed that they had heard about risk factors such as alcohol and obesity in relation to other conditions, specifically heart disease, however not in relation to breast cancer.

*“I was personally unaware of the changes regarding the weight gain in relation to the hormone changes. I think that this is a piece of information that is underestimated. At least for me it was not known. I’ve never heard of the relation with how you put on weight and the stage of your life in relation to the chances of developing breast cancer. It was more just like a generic one in eight women will develop cancer in their lifetime, breast cancer. So, for the me the striking point was the weight gain and the BMI index.”* (FG3, 40–49 yrs).

*“I thought it was BMI and alcohol – well more waist circumference was more to do with heart disease, not breast, or the chance of getting breast cancer.”* (FG 8, 60–74 yrs).

Upon digesting the information, some women were left in what they described as being almost a state of shock about this new, and for some women, confronting information.

*“This has really shocked the life out of me. I don’t want to be at risk of getting breast cancer.”* (FG8, 60–74 yrs).

*“I’m just shocked about the alcohol. I’m shocked. I’m shocked.” (FG4, 50–59 yrs)*.

Women tended to be more vocal in their responses and the discussion that they felt was most relevant to their situation. If a woman felt she drank alcohol more often (e.g. one or two glasses with dinner every night) or had gained weight in recent years, the information felt more personal and she focused on those statistics more than others. These women also tended to have questions in relation to risk that were specific to their lifestyle e.g. risk of binge drinking compared to a glass or two per night.

*“I feel I’m somewhat overweight. I do drink quite a bit of wine and I’m over 50, so it’s quite depressing. Yeah, it’s hard content to hear.”* (FG6, 50–59 yrs).

Some women expressed increased motivation to change their lifestyle behaviours after hearing about the risk factors.

*“I’ve put on quite a lot of weight, but that’s due to inactivity … It’s kind of making me feel like I should do something about it. I have lost a bit of weight, but it seems like I should do some more*.*”* (FG8, 60–74 yrs).

However, there were a few women also expressed reluctance to change their lifestyle behaviours after learning about this information, particularly in regard to alcohol consumption. These women acknowledged the lifestyle risk factors, but balanced the information with their personal preferences for quality of life and lifestyle.

*“There’s lots of things that you can’t change and you just have to – as I said, you’ve got to live a life and you can be aware of the things like smoking. I wouldn’t smoke. But I think by having – like certain amounts of risk that you’re willing to take to just have an enjoyable life.”* (FG9, 60–74 yrs).

Overall, women discussed that this information would allow them to make better informed decisions about their health. Of the few women who had heard about various modifiable risk factors previously, they acknowledged that this information was not always in the public space e.g. in mammography screening settings and that you often had to actively look for it.

*“You’ve got to do a bit of digging around and reading yourself to access what you want to know. It’s not always out there in the public space.”* (FG9, 60–64 yrs).

### Communicating important but there are important caveats to consider

#### Preference for sources of information vary

Women overwhelmingly felt that information about breast cancer risk factors, in particular potentially modifiable risk factors, should be communicated more broadly to women in the Australian community, particularly to women around the age of menopause. Although, it was also noted that this information should even be provided at an earlier age.

*“You can’t just wait until you’re 40, 50, even 30. It has to be something that us women, girls are aware since an earlier age. And just for the overall benefit of it, not just for the breast cancer, but the modifiable risk factors are going to have such an important chain effect onto a variety of other aspects of our lives … the earlier the better.”* (FG3, 40–49 yrs).

Women had varying preferences for sources of communication about breast cancer risk information. Many women, particularly those in the 40–49 and 50–59 years focus groups, identified social media as a way of engaging in information about breast cancer risk factors, particularly those who felt that they would not seek this information out otherwise e.g., women who did not have a family history of breast cancer or any other known risk factors.

*“But I think that some sort of government sponsored information campaign on social media – maybe a campaign to target people who perhaps wouldn’t otherwise access that information.”* (FG5, 50–59 yrs).

*“Sometimes, I do click on some ads on Facebook, so that might be a way, if it was presented, if I was just scrolling anyway, I might stop and look at something if it was presented in quite an easy format.”* (FG6, 50–59 yrs).

Women also identified GPs as a trusted source of information. However, women also expressed that they found GPs were often rushed and had limited appointment time or availability, and that they rarely saw GPs for prevention. Women’s health clinics were instead suggested as a place for discussion and to ask questions, if they were widely accessible.

*“GPs are always important because the credibility of the source of the information is probably important and GPs of course are trusted kind of person.”* (FG3, 40–49 yrs).

*“It’s hard to get in to see a GP these days. And they’re just, “What’s your issue, boom-boom-boom”, and you’re out.”* (FG5, 50–59 yrs).

*“So, if there was, like, a clinic or health women’s clinic where you could discuss anything, not just breast cancer, anything related to women”* ( FG2, 40–49 yrs).

Several women also preferred discussion-based methods (e.g., storytelling, conversations with peers).

*“I may stop and listen if it’s from a celebrity that I like and I know has been through breast cancer.”* (FG6, 50–59 yrs).

*“If you can relate to someone, or you see someone in advertising or someone telling their story and you can relate to them, like a mother with children and you’re a mother with children, you would be more likely to take notice because you can see yourself in their shoes.”* (FG4, 50–59 yrs).

There were a number of women who also discussed BreastScreen Australia as being a potential source of information. Women identified that this information could be potentially provided in the screening invitation information, appointment reminders or results letter, or at the time of the appointment. They felt that this would be a point in time when the topic of breast cancer would be on their mind and that they would be more likely to engage in the information, rather than just thinking only about the importance of screening in relation to breast cancer prevention.

*“But perhaps with that message that you get as a reminder, or I don’t know if they email, a link to the causes and risks of breast cancer could be included, so that you’d become aware of the risks.”* (FG9, 60–74 yrs).

*“Or while you’re at the mammogram place, there could be information there. Because it’s sort of on your mind when your getting a mammogram, it’s a prime time to get the messages.”* (FG8, 60–74 yrs).

A few women preferred interactive methods which they felt they would be more engaged in and would help make the information more personal.

*“I personally like some interactive, so you can put your own details or risk factors in and get some information about what your risk might be or – yeah, it makes it a bit more personalised, I guess.”* (FG6, 50–59 yrs).

Other suggestions that were spontaneously mentioned included having brochures at GP offices and BreastScreen clinics or private screening clinics when receiving a mammogram; advertising in public domains (e.g., in public bathrooms, women’s stores); and having warning labels on alcohol.

#### A ‘cascade of information’ preferred

There was a strong preference across the focus groups that women wanted the information presented as a ‘cascade of information’ where they would be presented with a bit of information with details, links or resources to further information which they could source if preferred. Women wanted the initial information to be eye-catching, engaging and relatable, where possible.

*“Pictures that usually grab my attention. If there’s something like a link to a website, then you can go there and look for further info. But yeah, it’s definitely pictures and graphs that grab my attention.”* (FG1, 40–49 yrs).

#### Beware of ‘information overload’

Several women reported ‘information overload’ as a barrier to accessing and engaging with health information, particularly in the last few years with COVID-19 information.

A few women also discussed that hearing too much information about health has led them to disregard the information and advice.

*“There’s just so much information about so many different types of cancer, I tend to just be like, yeah, okay, whatever.”* (FG1, 40–49 yrs).

A few women also reported being confused by health messaging, due to the information changing over time, or the different information and advice from different health conditions.

*“And it’s confusing, because one health study will say “Yeah, red wine’s great for your heart, good for the brain” and now, yeah, you think, well, this one’s saying any alcohol is bad for the breast.”* (FG6, 50–59 yrs).

#### Importance of reaching all women in the community

Women identified needing a widespread approach in terms of the different methods and sources of information with a concern about culturally and linguistically diverse communities and women with lower health literacy.

*“I also want to say that there’s about a quarter of our population that may not be so fluent in English and the information needs to be in other languages and other formats, and we are all not all very literate in health literacy, health languages.”* (FG7, 60–64 yrs).

### Interest in various interventions however, worry about long-term compliance

There were several different interventions (e.g. mobile applications, personalised lifestyle change plans, GP conversations/advice, motivational messages) that women showed interest in however, long-term compliance was discussed as a point of concern for women. They felt that often these types of interventions are not sustainable. Peer support was brought up by many women as a preferred lifestyle intervention that may be more sustainable. They felt that this would help hold them accountable. A few women in the older age groups wanted personalised coaching for the same reason.

*“We may as a generation tend to go towards that support group style and keeping each other accountable and motivating each other and supporting each other in triumphs and failures as well.”* (FG3, 40–49 yrs).

*“Someone [professional] to hold you accountable I guess.” (FG9, 60–74 yrs)*.

The suggestion of mobile applications (apps) received mixed opinions. Some women preferred apps as they felt this was an easy and convenient intervention, while others did not due to various reasons (e.g., easy to ignore or enter false information, and too many apps in their phones). For women who preferred apps, there was no clear pattern in age distribution.

*“I would say the apps, because I’m doing the health apps by phone, and I’m really sort of sticking to it and trying to lose weight and keep fit. And I think that for me personally is the way to go*.” (FG9, 60–74 yrs).

*“I have used apps before and they worked when you’re putting in the true information. You can choose not to put in things like that, if you know you’ve eaten too much or drank too much.”* (FG6, 50–59 yrs).

Increased access to allied health services was mentioned by some women as a way of supporting health lifestyle changes, in particular in relation to exercise support.

*“I’d like to see more support through Medicare for allied health services.”* (FG7, 60–74 yrs).

### Quantitative questionnaire responses

Table 3 outlines the responses to the quantitative questions that women completed at the end of the focus group session. Women overwhelmingly believed that information about potentially modifiable risk factors for should be communicated to women (98%) and that the information presented during the focus groups made them feel informed (90.2%). The majority of women believed that the information made them feel differently about their personal risk of breast cancer (74.5%) and made them want to do something to modify their personal risk of breast cancer (68.6%). The top 3 sources of information to communicated breast cancer risk factor information were online sources (39.2%), BreastScreen (33.3%) and GPs (19.6%).

**Table 3:** Participant responses to quantitative questionnaire questions (n = 51)

Response	No. of participants, n (%)
Do you think information about potentially modifiable risk factors for breast cancer should be communicated to women?^a^ Yes No	50 (98.0) 0 (0)
How would you like to receive information about risk factors? Online information BreastScreen program GP Leaflet or brochure Other screening service Breast physician	20 (39.2) 17 (33.3) 10 (19.6) 2 (3.9) 1 (2.0) 1 (2.0)
Did the information make you feel differently about potentially modifiable risk factors for breast cancer? Yes No Unsure	40 (78.4) 7 (13.7) 4 (7.8)
Do you think this information has made you feel differently about your personal risk of breast cancer?^a^ Yes No Unsure	38 (74.5) 8 (15.7) 3 (5.9)
Do you think this information has made you want to do something about modifying your personal risk of breast cancer?^a^ Yes No Unsure	35 (68.6) 7 (13.7) 7 (13.7)
Overall, how does knowing this information make you feel?^b^ Informed Anxious No different Confused	46 (90.2) 6 (11.8) 2 (3.9) 0 (0.0)

^a^Data missing

^b^Participants could select multiple items

## Discussion

This qualitative focus group study of peri- or post menopausal women living in Australia found that general knowledge of risk factors for breast cancer is limited. Women highlighted the importance of breast cancer screening to prevent breast cancer, however had little to no knowledge about risk factors, particularly modifiable risk factors. Findings from this study are similar to those in previous Australian quantitative studies [22, 23] which demonstrate that women generally can identify family history as a risk factor [23] but have misconceptions when identifying modifiable risk factors [22]. Specifically, in this study information related to alcohol and obesity were largely unknown. Women discussed having heard this information for other conditions such as heart disease but perceived it to be quite shocking in terms of the magnitude of risk involved for breast cancer. This often-personalised shock reaction to the information has not been described in detail in previous studies. A recent interview study of South Australian women aged 45–64 years explored alcohol and also found that women were unaware of this information, but also questioned the messages and messenger of the information [24].

The findings highlight the need to educate women in the Australian community about risk factors for breast cancer more broadly. While it is important to target women around the age of menopause, when risk factors for breast cancer change, education at an earlier age was mentioned in the focus groups and has been proposed by experts in the field of breast cancer prevention as an area in which prevention efforts could be expanded [4, 25]. For example, alcohol consumption during adolescence has been shown to increase the risk of precursors for breast cancer [26], while higher fibre intake and increased physical activity can reduce the risk of breast cancer [27, 28]. Including this information in childhood and adolescent health education through schools or health campaign messaging targeting younger women, might be a potential avenue to begin to disseminate this information and make it more mainstream but would require appropriate development and evaluation.

Another specific time point worthy of attention for prevention efforts, which women in the focus groups raised, was during their breast screening process. Women discussed that information could be given alongside their screening invitation and information or results letter and/or at the time of their appointment. They believed that this was a time when breast cancer would be top of mind and that they would be more likely to engage in this information. Additionally, when directly asked about information preferences in the final questionnaire, a third of the women chose the BreastScreen Australia program, as their preferred way of receiving information about risk factors for breast cancer. If this was to be implemented by the BreastScreen program careful consideration about how and when to best to provide this information to women would be needed.

Women in the focus groups overwhelmingly wanted to be told information on modifiable risk factors for breast cancer, however even within the age-range of women eligible for population-based screening, communication preferences varied widely. Findings, similar to previously mentioned work [24], highlight that a multi-pronged approach to communication is likely to be needed to reach women of all ages, socioeconomic status, and health and media literacy levels. However, it is known that knowledge of breast cancer risk factors or intention to modify behavioural factors does not guarantee actual lifestyle change, that there has generally been a lack of awareness among women of lifestyle behaviour change as a form of breast cancer prevention [6], and women do not feel confident about actions they can take to mitigate breast cancer risk [29]. Therefore, well developed and accessible interventions are needed to facilitate this. Despite worry about long-term compliance, women in this study showed interest and are generally supportive of interventions that may help them to reduce their risk of breast cancer. In a lifestyle intervention aiming to reduce breast cancer risk in women with a high risk of breast cancer, women expressed the desire for clear information on modifiable risk factors to improve motivation – in particular, how and why lifestyle factors increased breast cancer risk [30]. Furthermore, studies in breast and other cancers have demonstrated that providing lifestyle advice and interventions at cancer screening or related appointments is acceptable.[8, 31, 32] Thus, future research should further assess targeted communication preferences of women to motivate and sustain potential lifestyle change. Engaging with and involving key stakeholders, including screening service staff and clinicians, is also key in ensuring successful information dissemination and interventions targeting modifiable risk factors for breast cancer.[33].

To the best of the researchers’ knowledge this is the first Australian qualitative study which explores current knowledge, understanding and experience of various potentially modifiable risk factors for breast cancer, and views on current and future communication strategies for this information and potential intervention preferences. The focus groups were made up of a diverse sample of women from across Australia varying in education and age pre/post-menopause. The information that was presented to women was based on the best and most up-to-date evidence, and the presentation was developed by a multidisciplinary team, a consumer representative and was rigorously piloted tested prior to commencing.

The study may be limited by the complexity of some of the information presented to women. Participants’ understanding of the information was not testing although data indicated that most women engaged thoughtfully with the discussion. A formal assessment of women’s health literacy was also not collected and therefore, were not able to elaborate on how women’s health literacy might have impacted their overall responses. Conducting the focus groups with a small sample of women (4–6 in each group) face-to-face allowed for time to go through the information and to provide clarification where needed [34]. Furthermore, as there has been limited data on lifestyle interventions targeted at women in screening populations [35–37], a number of different potential lifestyle interventions that could be applied to reducing breast cancer risk generally were presented and discussed, also providing new insights that could assist current efforts in communicating and testing lifestyle-related interventions in screening programs [35–37].

## Conclusions

In conclusion, findings from this study highlight the limited awareness amongst women about lifestyle-related risk factors for breast cancer. It also highlights the need for more widespread community communication and education about these risk factors, in particular potentially modifiable risk factors such as alcohol consumption, postmenopausal obesity and physical activity. Building health literacy around these risk factors for breast cancer may support women to make informed decisions about their own breast health and potentially change behaviour or could motivate the uptake of effective prevention strategies. Furthermore, these findings are timely as breast screening programs in Australia and globally are beginning to evaluate the possibility of future risk-related screening for breast cancer which will provide an additional context for primary prevention. As planning of messaging and piloting of interventions regarding lifestyle prevention in breast cancer is needed now, gaining an understanding of women’s preferences for communication and forms of interventions is vital. Future studies can build on this work by further exploring some of the key issues identified, for example by tailoring communication about alcohol specifically in relation to breast cancer risk, or by canvassing knowledge, preferences and interest amongst younger age-groups and key stakeholders.

## Data Availability

The datasets generated and/or analysed during the current study are available from the corresponding author on reasonable request.
